# Hemidivisional vector planning to reduce and regularize irregular astigmatism by laser treatment

**DOI:** 10.1007/s00417-022-05604-x

**Published:** 2022-03-09

**Authors:** Noel Alpins, James K. Y. Ong, George Stamatelatos

**Affiliations:** 1 Department of Ophthalmology, Centre of Eye Research (CERA), The University of Melbourne, 160 Victoria Parade, East Melbourne, VIC 3002 Australia; 2 NewVision Clinics, Melbourne, Australia; 3 ASSORT, Cheltenham, Australia

**Keywords:** Vector planning, Irregular astigmatism, Corneal topographic astigmatism (CorT), Astigmatism reduction and regularization

## Abstract

**Purpose:**

To demonstrate how hemidivisional vector planning of refractive laser treatments of astigmatism can be used to directly address idiopathic corneal irregular astigmatism that has an asymmetrical, non-orthogonal bow tie topography appearance.

**Design:**

Case study.

**Methods:**

The cornea is conceptually divided into two hemidivisions along the flat meridian of the corneal topographic astigmatism (CorT), which means that each hemidivision will approximately correspond to one lobe of the asymmetric, non-orthogonal topographic bow tie. An astigmatism reduction treatment can then be planned separately for each hemidivision using the vector planning technique, based on both its two hemidivisional CorT measures and common manifest refractive cylinder. The remaining irregularity is then regularized, and the junctional zone smoothed across the flat meridian. The final intended treatment thus combines hemidivisional astigmatism reduction and regularization of the corneal astigmatism and spherical refractive error in one treatment application. This could be applied to LASIK, PRK, SMILE, and Transepithelial PRK procedures using Designer Cornea^®^ software.

**Results:**

A theoretical treatment profile is derived from an actual example of a cornea with idiopathic asymmetric non-orthogonal astigmatism. The three steps of the derivation are as follows: (i) astigmatism reduction through the use of the vector planning technique; (ii) regularization, and (iii) smoothing across the hemidivisional midline.

**Conclusions:**

Hemidivisional vector planning treatments could potentially both reduce and regularize asymmetric non-orthogonal astigmatism. These treatments can be systematically customized to account for qualitative and quantitative differences between the two corneal hemidivisions at the same time as correction of coexistent myopia or hyperopia.

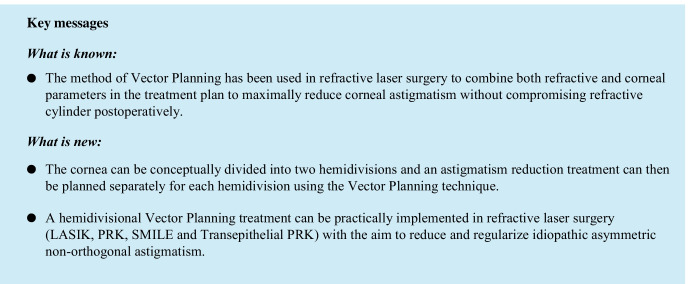

## Introduction

Many different paradigms for excimer laser refractive treatment can be used to address corneal irregularity. One of these approaches is to perform topography-guided ablations. The primary aim of topography-guided treatments is to directly smooth out corneal irregularities and target a desired aspheric shape; a secondary aim is to attempt to address spherocylindrical refractive error [1]. However, recent studies disagree on whether topography-guided ablations should treat the manifest refractive cylinder [2–5], the manifest refractive cylinder adjusted by the refractive effect of the smoothing [6–9], the topographically measured corneal astigmatism [10–12], or some compromise between the manifest refractive cylinder and the corneal astigmatism [13].

The disagreement about the amount and orientation of cylinder to be treated appears to be due in part to the types of eyes selected for each study. The mismatch between the corneal astigmatism and the manifest refractive cylinder (also known as the ocular residual astigmatism, or ORA [14]) may affect visual outcomes differently for different treatment targeting strategies [3, 12]. However, some studies are conservative in their selection of eyes, only selecting normal virgin eyes that have low preoperative ORA [2, 15, 16], others use normal virgin eyes with no ORA requirement [10, 11], and some specifically choose to focus on eyes with high preoperative ORA. Some studies even include eyes with irregular astigmatism [3, 17]. The substantially different populations of eyes make it very difficult to compare visual outcomes between studies.

Different studies also report different types of outcomes. Invariably, postoperative spherocylindrical refraction and corrected and uncorrected visual acuities are reported, but measures of higher order aberrations (HOAs) are only reported in some of the studies [10, 17], sometimes only indirectly through patient feedback about night vision disturbances like halos and glare [11]. Treatment strategies that only neutralize the manifest refraction aim to maximize the postoperative uncorrected visual acuity, while strategies that sphericize the cornea aim at reducing HOAs. It is therefore not surprising that proponents of either type of strategy at two extremes of options will emphasize those results that favor their preferred method.

The contrast between approaches of neutralizing the manifest refraction and sphericizing the cornea is not new. Vector planning (VP) [14] can be used to find an optimal balance between the conflicting two surgical goals, even in eyes with mild keratoconus [18]. Studies have shown that the postoperative manifest refractive cylinder from a VP treatment is comparable or lower than a pure refractive treatment, and the postoperative HOAs are also comparable or lower [19, 20]. The fact that the postoperative refractive cylinder appears to be paradoxically low is a corollary of the observation that VP treatments lead to a reduction in ORA [18, 20]. An alternative method (mutual comparative analysis) adjusts the manifest refractive cylinder to be closer to the corneal astigmatism. This method also results in excellent postoperative uncorrected visual acuity and refractive outcomes [13].

In this paper, we show how a hemidivisional vector planning treatment [21] can be practically implemented as a treatment for idiopathic asymmetric non-orthogonal astigmatism. This is a more customizable version of a previously proposed method [22], where the treatment profile was calculated as the sum of a regularization treatment (which targets orthogonal astigmatism) and a reduction treatment (which reduces the resulting orthogonal astigmatism across the whole cornea). The new method allows for the balance between refractive cylinder and corneal astigmatism to be different in each of the two corneal hemidivisions for LASIK, PRK, SMILE, and Transepithelial PRK procedures. Such a treatment profile still aims for a reduced amount of symmetric orthogonal astigmatism and targets a spherical equivalent of zero. We analyze the effect of such a treatment on corneal HOAs. We also consider the effects of subsequent regularization treatment and midline profile smoothing, both of which are generally necessary in real-world treatment profiles.

## Materials and methods

Hemidivisional vector planning for astigmatism reduction and regularization was first described by Alpins [21] long before there was any possibility of implementing it as part of a laser refractive treatment. We give a brief introduction to the basic concepts here before showing how this technique can be combined with regularization and smoothing components into one summated treatment that enhances the optical performance of the technique and allows it to be implemented practically.

### Vector planning

The vector planning method [14] is used to calculate a corneal refractive laser treatment of astigmatism that balances the often-conflicting goals of zero corneal astigmatism and zero refractive cylinder in the common situation where there is a preoperative mismatch. The idea is to represent the corneal astigmatism (magnitude @ steep meridian) and the refractive cylinder (magnitude X positive cylinder axis) on a double angle vector diagram at twice their orientations but with the same magnitudes. Then, the straight line joining these two double angle measures (also known as the *ORA line* [14]) defines the set of treatments that provide optimal reduction of both the corneal astigmatism and the refractive cylinder. At one extreme of this ORA line, the treatments emphasize reducing corneal astigmatism, while at the other extreme, the treatments emphasize reducing refractive cylinder. In normal corneas with low levels of regular astigmatism, it is often appropriate to choose a 60% emphasis on reducing refractive cylinder [19] rather than conventional 100% carried out in the vast majority of refractive laser treatments. However, the appropriate emphasis may change with increasing levels of ORA, or if various target orientations are preferred to favor corneal or refractive priorities [18].

### Vector planning method applied to corneal hemidivisions

The vector planning method can be easily applied to corneal hemidivisions with a significant adjustment: a topographical measure of hemidivisional corneal astigmatism is used instead of whole-of-cornea corneal astigmatism. In the example in this paper, we use the flat meridian of the corneal topographic astigmatism (CorT) to conceptually divide the cornea into two hemidivisions. Each corneal hemidivision then has a corresponding hemidivisional corneal topographic astigmatism (hemiCorT) [23]. Our strong preference is that each of the hemidivisional corneal astigmatism measures should be based on total corneal power. This allows for the effect of the posterior cornea on corneal astigmatism to be taken into account [24]. Note, however, that any reasonable measure of hemidivisional corneal astigmatism may be used with the method described in this paper.

We use the flat meridian of the CorT to divide the cornea. This allows for full unmodified treatment of steep areas of the cornea, which can be relatively flattened by laser treatment. The smoothed transition zone of the treatment profile (see below) then coincides roughly with flat areas of the cornea.

In this paper, we use manifest refractive cylinder, but a measurement of second-order ocular wavefront cylinder can also be employed. It may also be possible to perform two distinct ocular wavefront cylinder measurements through the halves of the pupil that correspond to the two corneal hemidivisions if this was measured.

### Reduction

For each corneal hemidivision, the hemidivisional corneal astigmatism measure and the refractive cylinder measure are represented on a double angle diagram. The straight line between these two measures on a double angle diagram (which is equivalent to the hemidivisional version of the ORA line) represents the possible combinations of hemidivisional corneal astigmatism and refractive cylinder that should be treated in order to allow both to be reduced optimally. These combinations are parameterized by the relative refractive or corneal emphasis of the treatment, where a 100% refractive and 0% corneal emphasis corresponds to a conventional refractive treatment that targets zero refractive cylinder, with the resulting corneal astigmatism neutralizing the preexisting ORA.

In the case of corneal asymmetric non-orthogonal bow tie astigmatism, it is likely that a different emphasis would be used in each corneal hemidivision. If one hemidivision had a lot more corneal astigmatism than the other, then it would make sense to place greater emphasis on a reduction of corneal astigmatism for the hemidivision with the greater amount of astigmatism, while placing greater emphasis on a reduction of refractive cylinder for the hemidivision with the lesser amount of astigmatism. The orientation of the two resultant target corneal astigmatisms may also influence the choice of emphasis. If the targeted corneal astigmatism was at an unfavorable orientation such as oblique or against-the-rule, then more emphasis might be given to corneal astigmatism reduction.

### Regularization

The two hemidivisional treatments are planned separately. If the initial astigmatism was irregular, it is unlikely that the reduction treatments will result in regular symmetric orthogonal corneal astigmatism. Thus, it will be necessary to regularize the resulting decreased but irregular corneal astigmatism with an astigmatism-neutral regularization component. The overall amount of astigmatism for the whole cornea should equal the double angle vector average of the two hemidivisional astigmatisms. Thus, the regularization step is simply to use this double angle vector average as the ultimate astigmatism target for each corneal hemidivision.

### Smoothing

The hemidivisional treatments that result from vector planning reduction and regularization will probably not be symmetrical about the midline raphe that separates the corneal hemidivisions. This means that the raw treatment profile would have a step discontinuity at the midline junction between hemidivisions. This would create both physiological and optical problems. To avoid such a step discontinuity, it is necessary to smooth the profile of the hemidivisional functions.

An appropriate smoothing procedure should satisfy a number of constraints: (i) the resulting profile should be continuous to avoid cliffs, (ii) the first and second derivatives with respect to polar angle of the resulting profile should be continuous to avoid sharp edges and artificial microstructure, and (iii) the Zernike decomposition of the profile should remain unchanged to ensure predictability of the final refraction.

For this paper, we smooth the profile up to 45 polar degrees on either side of the midline raphe. We use a parameterized convex combination of the two hemidivisional profiles. If the two hemidivisional profiles are f(θ) and g(θ), with a midline at polar angle α, then the smoothed profile is λ(θ)f(θ) + (1—λ(θ)g(θ)). We use the parameterization:

$$\lambda (\theta )=\left\{\begin{array}{cc}0,& \theta \in \left[\alpha -\frac{3\pi }{4},\alpha -\frac{\pi }{4}\right]\\ \frac{{\int }_{\alpha -\pi /4}^{\theta }{\left(\varphi -\left(\alpha -\frac{\pi }{4}\right)\right)}^{2}{\left(\varphi -\left(\alpha +\frac{\pi }{4}\right)\right)}^{2}d\varphi }{{\int }_{\alpha -\pi /4}^{\alpha +\pi /4}{\left(\varphi -\left(\alpha -\frac{\pi }{4}\right)\right)}^{2}{\left(\varphi -\left(\alpha +\frac{\pi }{4}\right)\right)}^{2}d\varphi },& \theta \in \left(\alpha -\frac{\pi }{4},\alpha +\frac{\pi }{4}\right)\\ 1,& \theta \in \left[\alpha +\frac{\pi }{4},\alpha +\frac{3\pi }{4}\right]\\ \frac{{\int }_{\alpha +5\pi /4}^{\theta }{\left(\varphi -\left(\alpha +\frac{3\pi }{4}\right)\right)}^{2}{\left(\varphi -\left(\alpha +\frac{5\pi }{4}\right)\right)}^{2}d\varphi }{{\int }_{\alpha +5\pi /4}^{\alpha +3\pi /4}{\left(\varphi -\left(\alpha +\frac{3\pi }{4}\right)\right)}^{2}{\left(\varphi -\left(\alpha +\frac{5\pi }{4}\right)\right)}^{2}d\varphi },& \theta \in \left(\alpha +\frac{3\pi }{4},\alpha +\frac{5\pi }{4}\right)\end{array}\right.$$

**Fig. 1:**
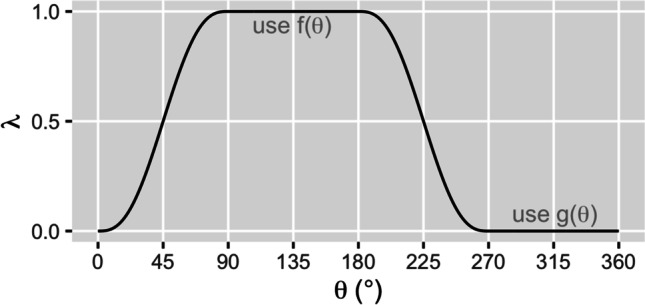
Plot of parameterization λ(θ) for the example where the hemidivisional midline is at 45°. This parameterization is designed to result in a smooth transition from f(x) to g(x) and back. When λ(θ) = 1, the smoothed profile is exactly f(θ). When λ(θ) = 0, the smoothed profile is exactly g(θ)

This parameterization λ(θ) is a stepwise quintic polynomial that satisfies the smoothness constraints. It produces a rotationally antisymmetric smoothing that does not affect the Zernike decomposition of the profile. The shape of λ(θ) (see Fig. 1) exhibits the smooth transition of the convex combination from one hemidivisional profile to the other.

Other methods of smoothing like splines would also be suitable as long as they do not change the Zernike decomposition of the profile.

## Results

**Fig. 2:**
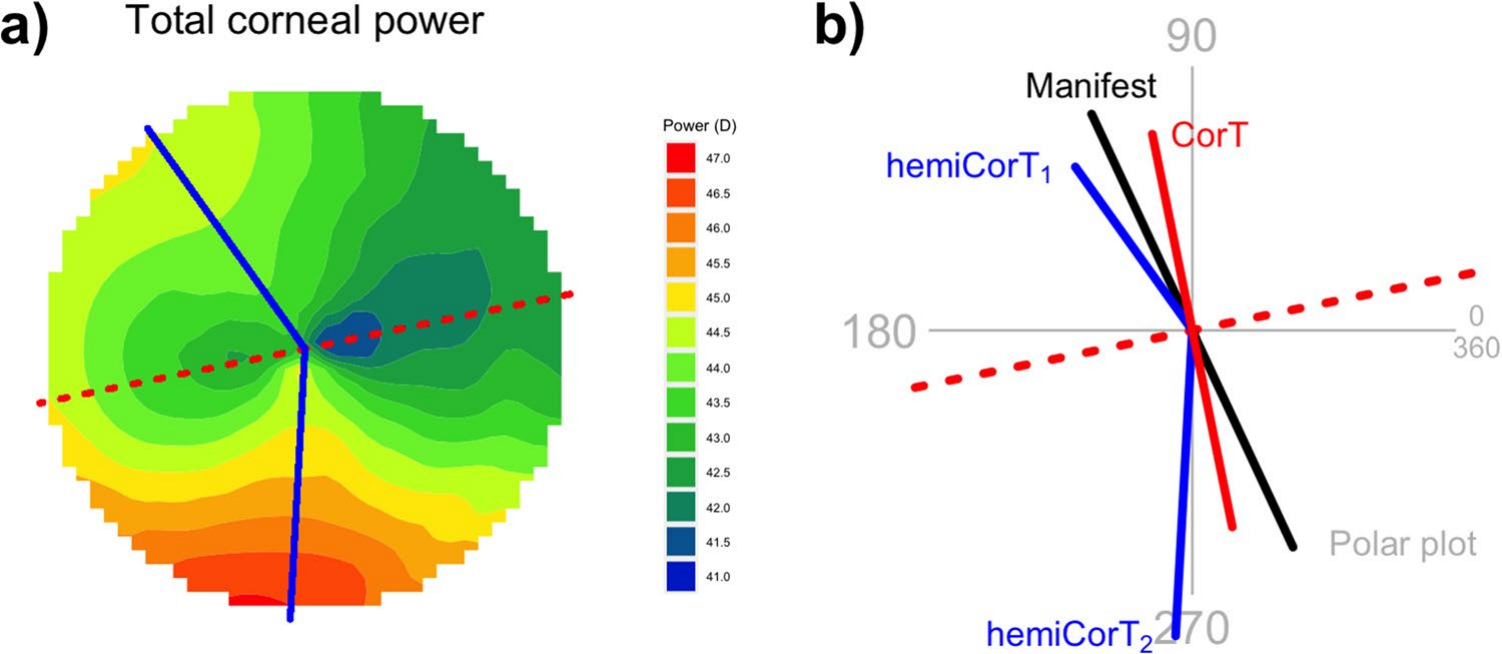
**a** Total corneal power map. The total corneal power data shown is from the central 7 mm zone. The dashed line at 12 degrees is the hemidivisional boundary. The solid lines at 126 degrees and 267 degrees represent the steep meridian of the hemiCorTs. **b** Polar plot summarizing relevant corneal and refractive parameters

In this section, we work through an example to show how the methodology described above is applied. We use the same example that was described in a previous paper [22] so that the results can be compared directly. This includes the final treatment profile and Zernike decomposition, but also the expected manifest refractive cylinder, CorT, and remaining corneal irregularity. The raw data comes from an eye with asymmetric, non-orthogonal astigmatism, characterized by inferior steepening. The total corneal power data was measured with a CSO Sirius tomographer (Costruzione Strumenti Oftalmici, Firenze, Italy). Total corneal power is shown as a map in Fig. 2a. The CorT is calculated from an annulus of between 2.0 and 5.2 mm diameter for this tomographer [24]. CorT is 1.90 D with a steep meridian at 102 degrees. The hemidivisional boundary is at 12 degrees. The superior hemiCorT is 1.90 D with a steep meridian at 126 degrees and the inferior hemiCorT is 2.90 D with a steep meridian at 267 degrees. The manifest refraction at the corneal plane is − 0.99/+2.26 × 115 degrees. These values are shown on Fig. 2b.

The superior hemidivision has an amount of hemidivisional corneal astigmatism that is close to the manifest cylinder in both magnitude and orientation; this corresponds to a hemidivisional ORA with a magnitude of 0.86 D. This value is only marginally larger than average for normal eyes—previous studies have an ORA average magnitude of 0.81 D [14] and 0.73 D [25]. When the ORA has this magnitude, it is appropriate to treat with an emphasis towards the refraction because the treatment is expected to leave only a moderate amount of postoperative corneal astigmatism. We select an emphasis of 80% refractive, 20% corneal.

The inferior hemidivision has an inferior steepening that is quite distinctly different from the manifest cylinder in both magnitude and orientation; here, the hemidivisional ORA has a magnitude of 2.47 D, which is large. When there is a large ORA, it is appropriate to select a treatment that more directly targets corneal sphericity for the corneal hemidivision [14]. In this worked example, we select an emphasis for the inferior hemidivision of 50% refractive, 50% corneal, which matches high-ORA treatments described previously [18].

**Fig. 3:**
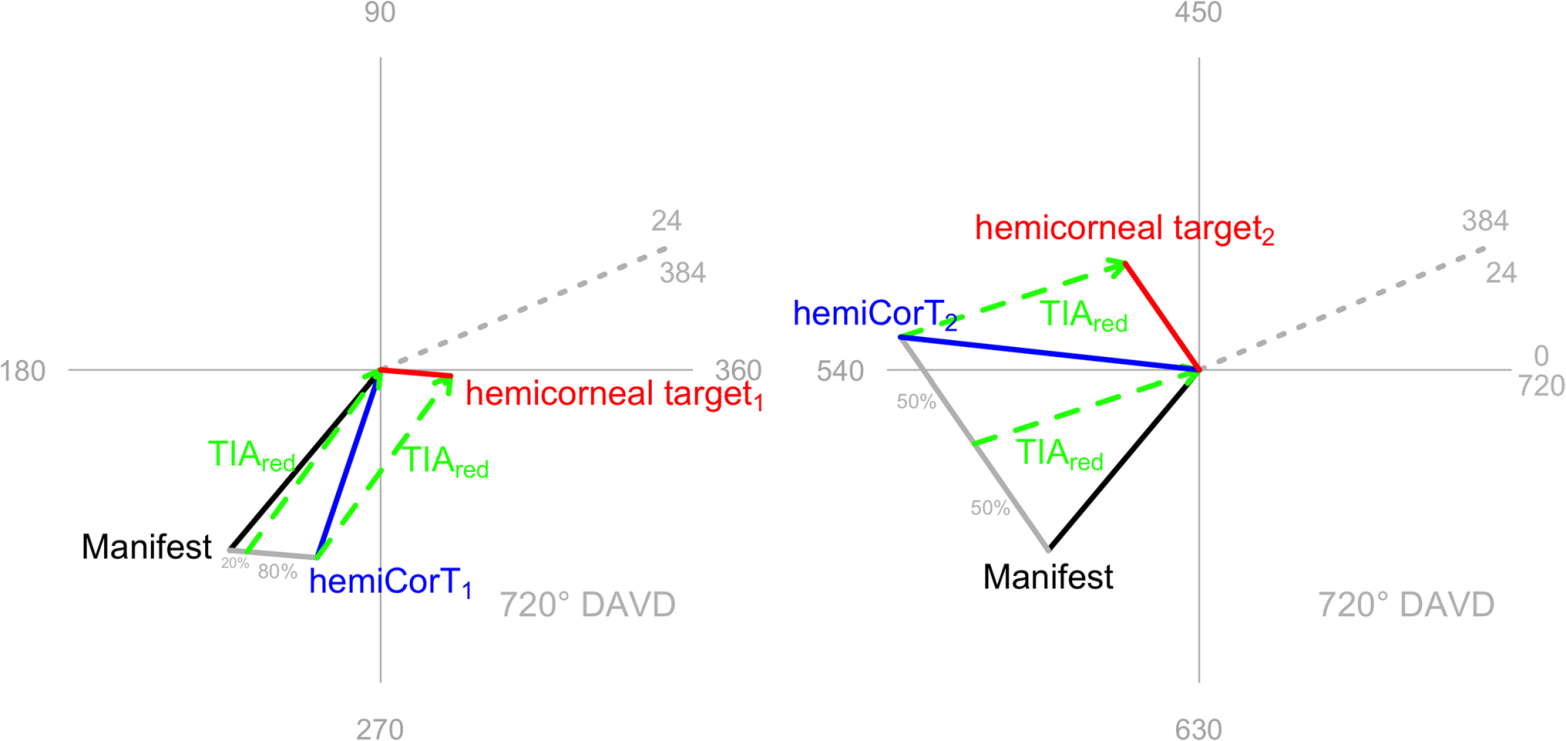
Hemidivisional vector planning for both corneal hemidivisions. The hemidivisional vector planning treatments (TIA_red_) are shown for each corneal hemidivision. **a** Vector planning with an emphasis of 80% refractive and 20% corneal is appropriate for the superior corneal hemidivision because the magnitude of the ORA is similar to that found in normal eyes. **b** The inferior corneal hemidivision has a large ORA, which is better addressed by vector planning treatment with an emphasis of 50% refractive and 50% corneal

Figure 3 shows both hemidivisional vector planning treatments on double angle vector diagrams (DAVDs). The superior hemidivision has a vector planning target induced astigmatism (TIA) of 2.17 D Ax 27, leading to a hemidivisional corneal target of 0.68 D with a steep meridian at 178 degrees. The inferior hemidivision has a vector planning TIA of 2.28 D Ax 9, leading to a hemidivisional corneal target of 1.25 D with a steep meridian at 62 degrees.

Note that we do not explicitly consider the hemidivisional corneal target meridian here because the regularization component of the treatment (see below) will change these again.

**Fig. 4:**
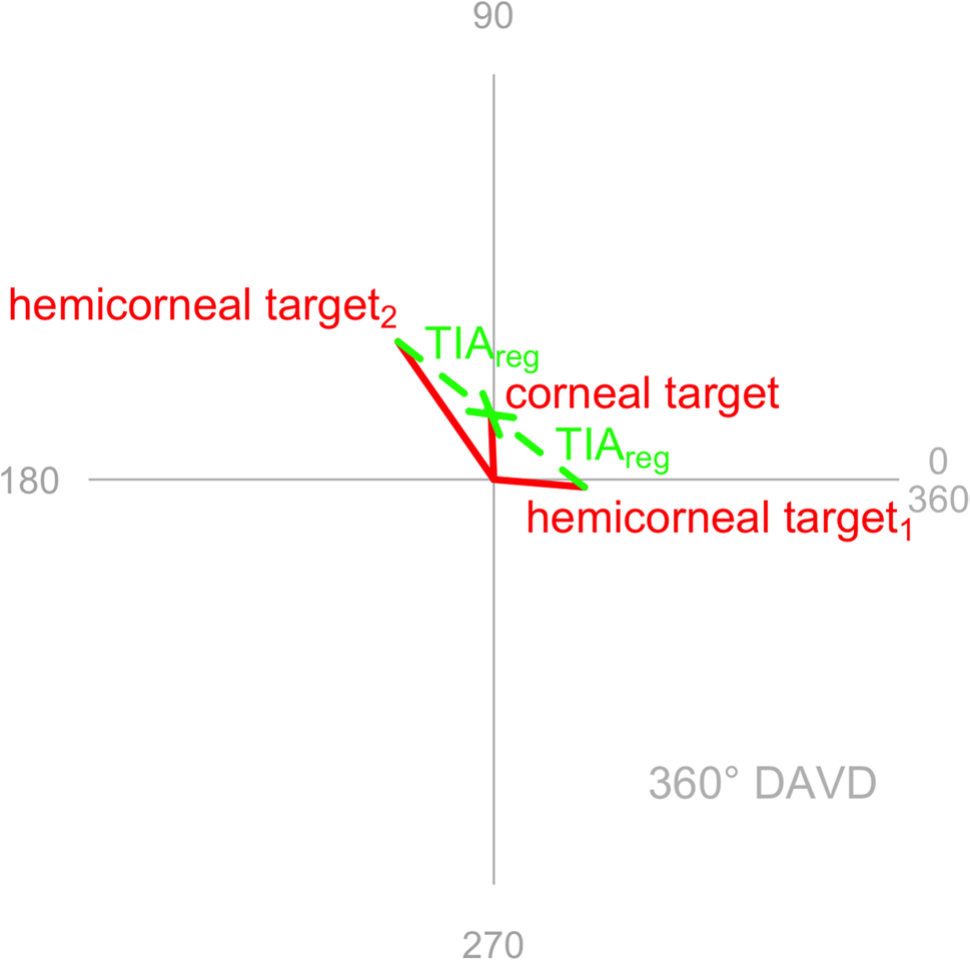
Regularization that achieves the same corneal target in both corneal hemidivisions

The two hemidivisional corneal targets are different in magnitude and orientation. The next required planning step is to regularize the treatment so that the two hemidivisional corneal targets end up being the same (overlying each other, though numerically 360 degrees apart on a DAVD). The regularized corneal target is the summated vector mean of the two hemidivisional corneal targets from the vector planning reduction step. Figure 4 shows the two regularization TIAs (0.88 D Ax 71 and 0.88 D Ax 161) that are required to achieve the corneal target of 0.48 D with a steep meridian at 46. Note that the two regularization TIAs will always have the same magnitude and will be 90 degrees apart (or equivalently 180 degrees apart on a DAVD).

**Fig. 5:**
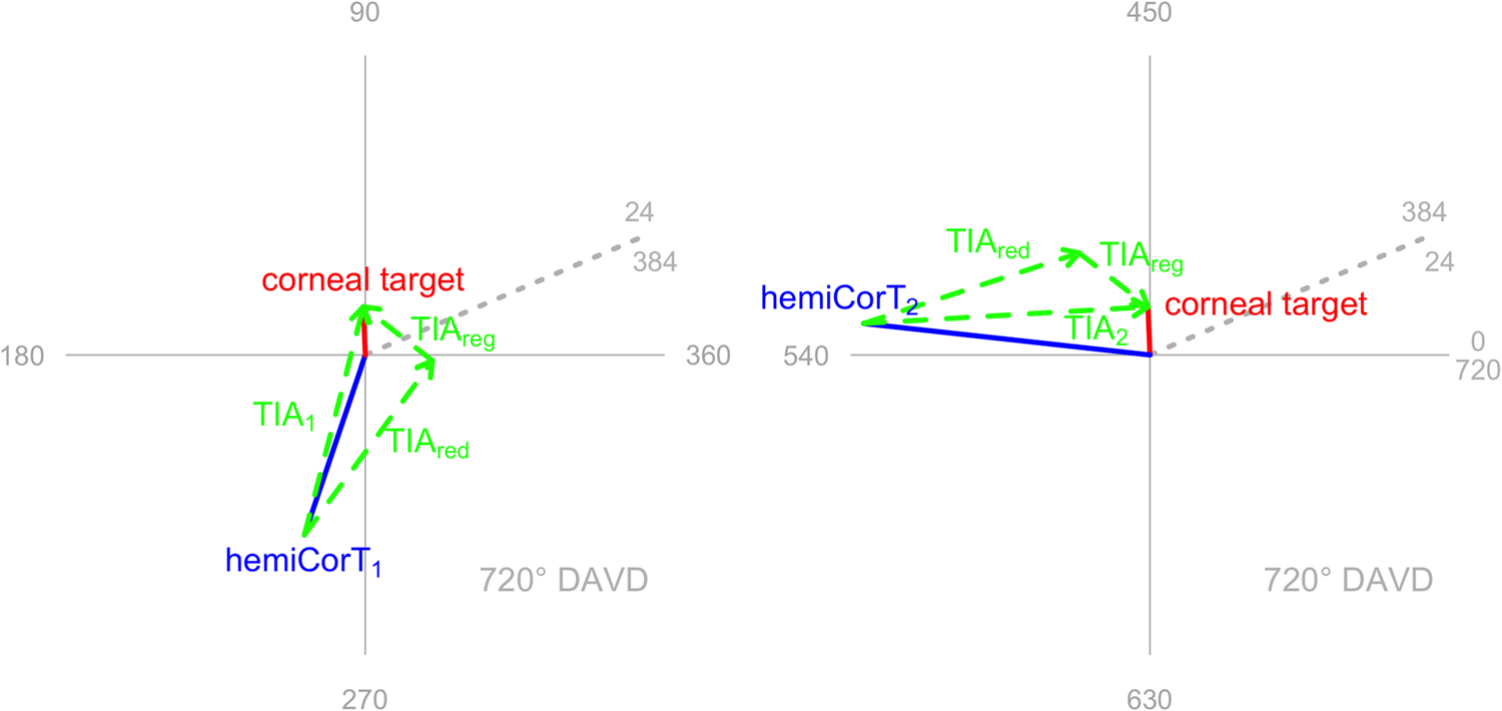
Combined treatments (TIA_1_, TIA_2_) incorporating the vector planning reduction (TIA_red_) and the regularization (TIA_reg_) treatments for each corneal hemidivision. Both combined hemidivisional treatments aim for the same corneal target (even though they are numerically 360 degrees apart on a DAVD)

For each corneal hemidivision, the vector planning reduction and the regularization treatments are combined into an overall single step hemidivisional treatment (see Fig. 5). The combined hemidivisional TIA for the superior hemidivision is 2.36 D Ax 38, while the combined hemidivisional TIA for the inferior hemidivision is 2.86 D Ax 2.

**Fig. 6:**
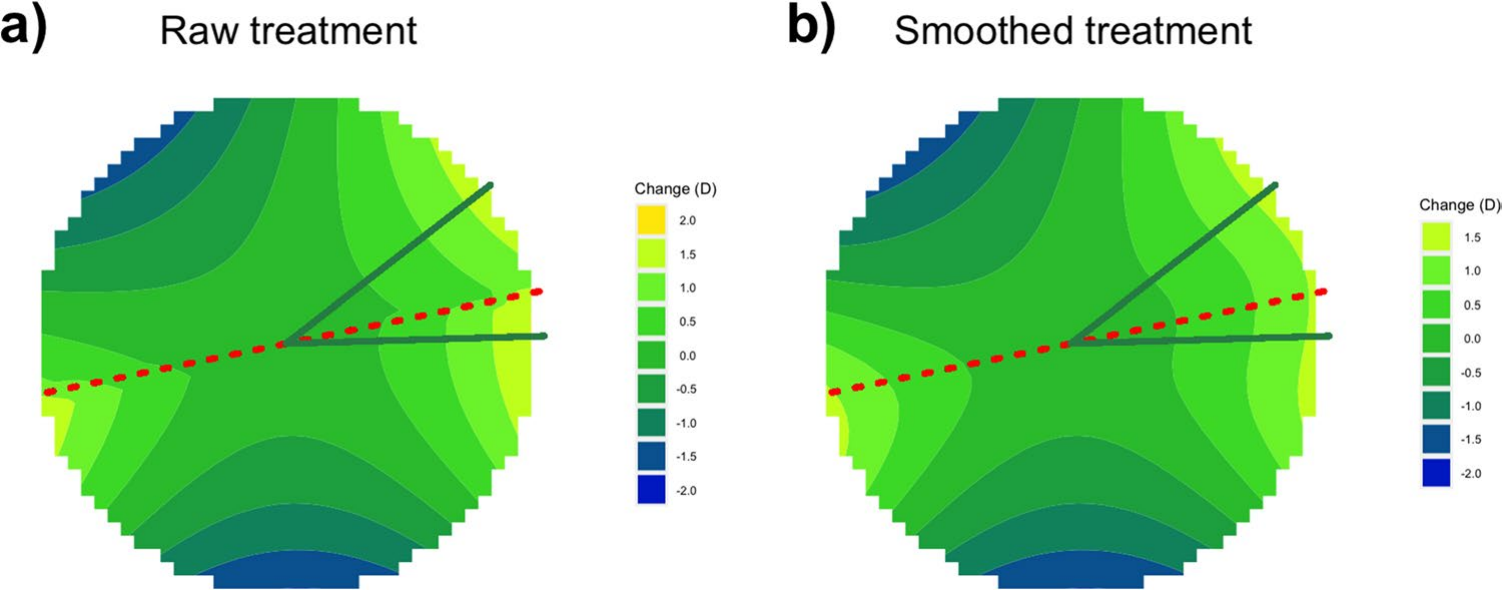
**a** Raw treatment profile, which comprises the two combined hemidivisional treatments. The hemidivisional TIA axes (superior 38 degrees, inferior 2 degrees) are superimposed in green. Note the step discontinuity in this treatment profile at the hemidivisional boundary. **b** Smoothed treatment profile. There is now no more step discontinuity at the hemidivisional boundary

The raw unsmoothed treatment profile is shown in Fig. 6a, with the hemidivisional TIA axes superimposed. To deal with the step discontinuity at the hemidivisional boundary, we apply the smoothing methodology described in the “Materials and methods” section. Figure 6b shows the resulting smoothed treatment profile.

**Fig. 7:**
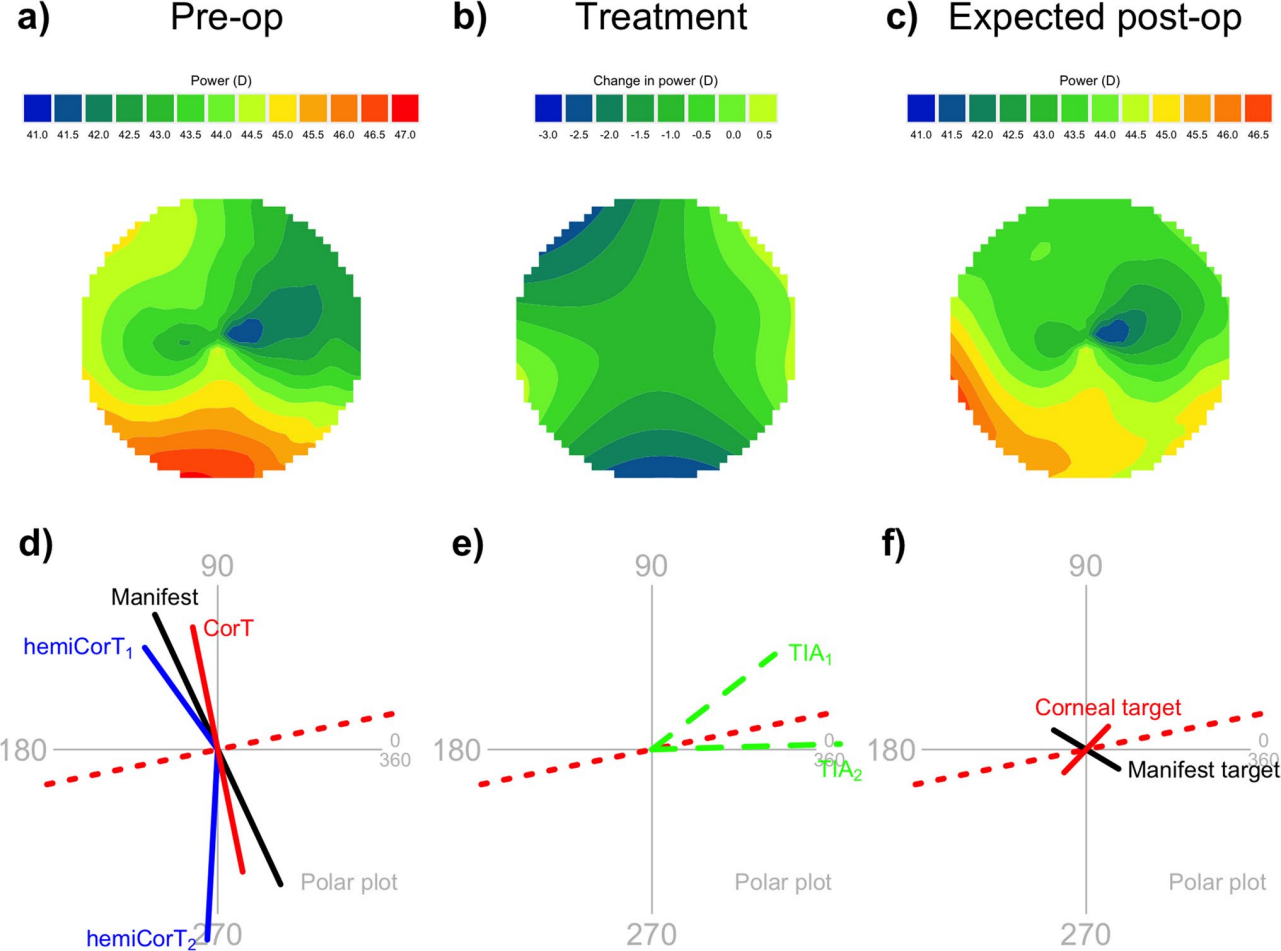
Summary of the effect of the smoothed combined treatment on the total corneal power map. This particular treatment has a superior refractive emphasis of 80% and an inferior refractive emphasis of 50%. **a** Preoperative total corneal power map, central 7 mm zone, same as Fig. 2a. **b** Smoothed treatment profile, same as Fig. 6b. **c** Expected postoperative total corneal power map. **d** Preoperative summary polar plot, same as Fig. 2b. **e** Hemidivisional TIAs shown on a polar plot. **f** Expected postoperative manifest cylinder and corneal astigmatism targets

Figure 7 summarizes the overall effect of the treatment. The expected postoperative result (Fig. 7c) is obtained by adding the smoothed treatment (Fig. 7b) to the original total corneal power map (Fig. 7a). In practice, there may be an associated spherical component added to the treatment, for example, that converts the treatment into one that only flattens, not steepens. Such a spherical component would then shift the vertical scale by a constant amount.

The expected postoperative result does not look entirely regular because astigmatic treatments do not have the capability to change the central corneal power to any great extent. However, the resulting profile is markedly more symmetric than the preoperative profile. The overall astigmatism in the map has been regularized by way of inducing a peripheral steepening (at about 200 degrees) that is opposite the central flat area (at about 20 degrees). The total amount of corneal astigmatism is greatly reduced (compare Fig. 7d and f).

The amount of corneal irregularity, as quantified by the magnitude of the topographic disparity [21], is reduced by the hemidivisional astigmatism reduction component from an original amount of 3.10 to 1.76 D. This is further reduced to zero by the astigmatism regularization component.

A Zernike decomposition of the smoothed treatment shows the following changes in addition to the reduction of second-order astigmatism:

1. A reduction in first-order components (tilt, tip),
2. A reduction in third-, fifth-, and seventh-order coma,
3. An increase in third-order trefoil and fifth-order pentafoil, and
4. An increase in fourth-order astigmatism.

**Fig. 8:**
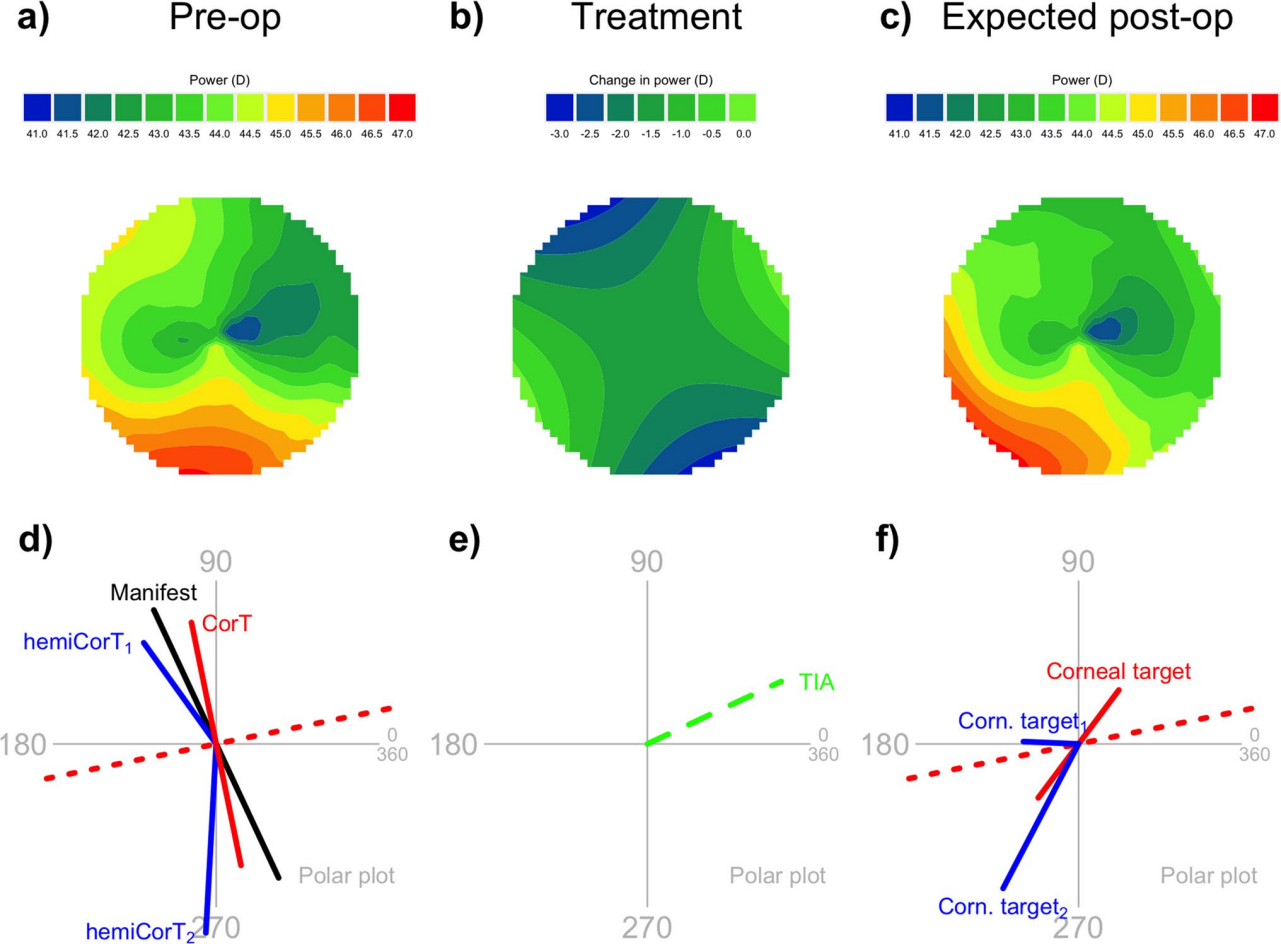
Summary of the effect of pure refractive cylinder treatment on the total corneal power map. **a** Preoperative total corneal power map, central 7 mm zone, same as Fig. 2a. **b** Pure refractive cylinder treatment profile. **c** Expected postoperative total corneal power map. **d** Preoperative summary polar plot, same as Fig. 2b. **e** Whole-of-eye TIA shown on a polar plot. **f** Expected postoperative whole-of-eye (red) and hemidivisional (blue) corneal astigmatism targets. The expected postoperative manifest cylinder is zero, but the corneal astigmatism remains asymmetric and non-orthogonal

To allow comparison of the proposed method with a standard refractive cylinder treatment, we show the effects of a standard treatment in Fig. 8. The expected postoperative profile (Fig. 8c) remains highly asymmetric and exhibits a large amount of corneal astigmatism overall (Fig. 8f).

**Fig. 9:**
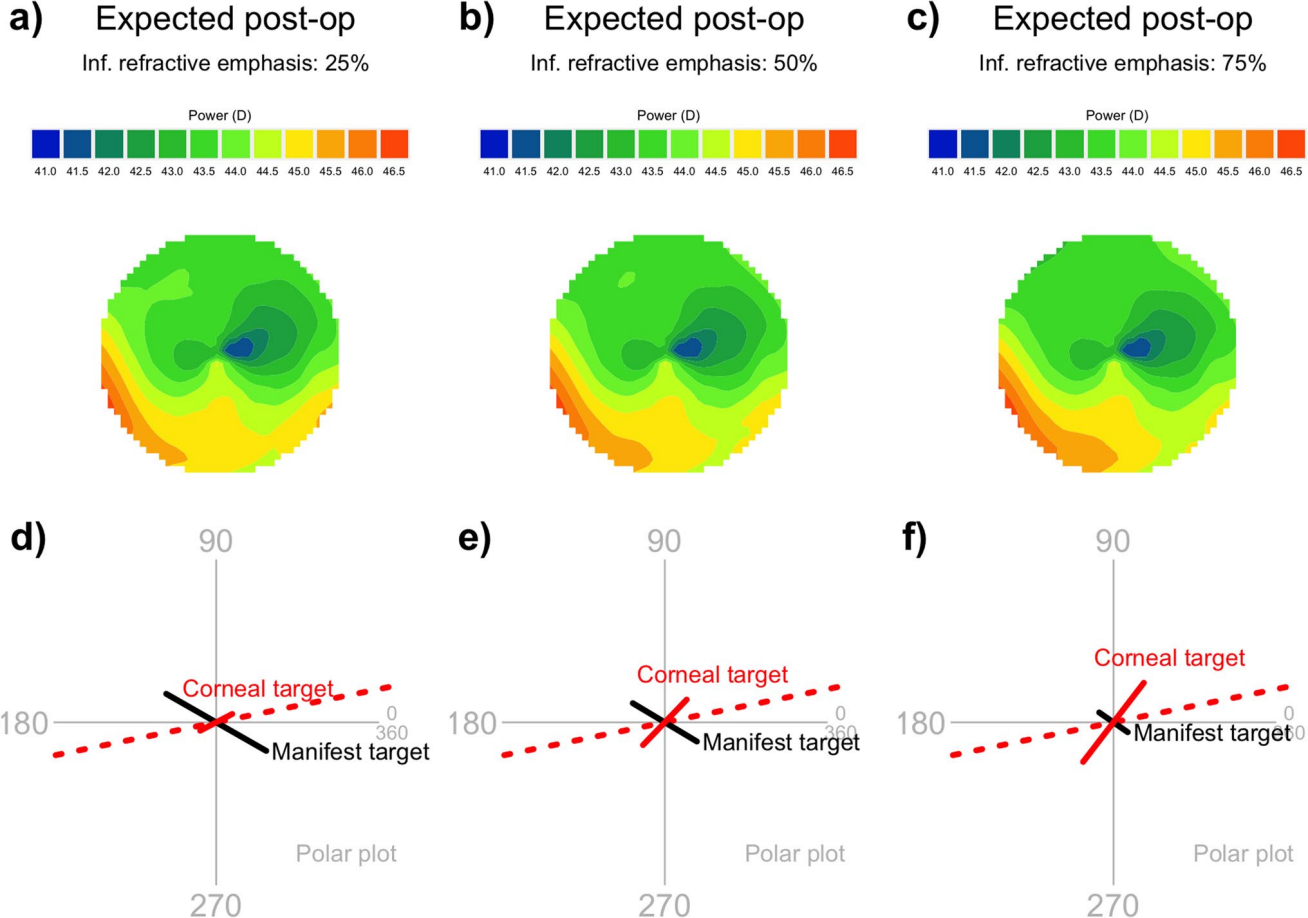
Comparison of expected postoperative results with different refractive emphases. All figures share a superior refractive emphasis of 80%. The three columns have varied inferior refractive emphases: from left to right, 25%, 50%, and 75%, respectively. The top row shows the expected postoperative total corneal map, and the bottom row shows the manifest cylinder and corneal astigmatism targets. As the refractive emphasis increases, the magnitude of the corneal astigmatism target increases and the magnitude of the manifest refractive cylinder target decreases

In Fig. 9, we show how a change in the refractive emphasis for one corneal hemidivision will affect the expected postoperative results.

- A low refractive emphasis (Fig. 9a and d) prioritizes corneal sphericity over low manifest refractive cylinder. In our example, a 25% inferior refractive emphasis targets low corneal astigmatism (0.28 D @ 28) and moderate refractive cylinder (0.88 D × 150).
- A medium refractive emphasis (Fig. 9b and e) balances refractive cylinder against corneal sphericity. In our example, a 50% inferior refractive emphasis targets almost equal amounts of corneal astigmatism (0.48 D @ 46) and refractive cylinder (0.57 D × 149).
- A high refractive emphasis (Fig. 9c and f) prioritizes low manifest refractive cylinder over corneal sphericity. In our example, a 75% inferior refractive emphasis targets moderate corneal astigmatism (0.76 D @ 52) and low refractive cylinder (0.27 D × 145).

By altering the refractive emphases in each corneal hemidivision, it is possible to shift the balance between the competing requirements of unaided visual acuity, which depends on manifest refractive cylinder, and visual quality, which depends on the extent of corneal irregularity.

## Discussion

The method described in this paper allows a surgeon to plan a refractive laser treatment that addresses idiopathic corneal irregular astigmatism with an asymmetrical, non-orthogonal bow tie topography. The intended treatment is customized differently for each corneal hemidivision, and the expected postoperative corneal astigmatism is reduced, symmetrical, and orthogonal. The postoperative manifest refraction is expected to be predictable because of the similarity of the treatment profile to standard refractive treatment profiles.

This method allows for greater customization than the method previously proposed by Alpins et al. in 2018 [22]. Different vector planning emphases are possible in each corneal hemidivision. This provides many more treatment options to fine-tune the outcome. The treatment derived in the 2018 paper is a special case of the possible treatments outlined in this paper, where the vector planning emphases for the two corneal hemidivisions are equal. It is not surprising that the Zernike decompositions for the two methods are similar, since the basic shapes of the treatment profiles are very similar. The main differences between the treatments are mostly due to the different vector planning emphases and their effects on the magnitudes of the hemidivisional treatments.

It has previously been demonstrated that regularization of an irregular cornea has the potential to improve best corrected visual acuity [26] and reduce undesired visual disturbances that are often associated with higher order aberrations [17]. It has also been shown that vector planning, which considers both corneal and refractive parameters, produces good visual outcomes [20], even in cornea with mild keratoconus [18]. The process of hemidivisional vector planning combines both astigmatism reduction and regularization in one pre-prepared treatment plan, with the possibility of concurrent change of spherical equivalent. Further subdivision of the cornea (e.g., into quadrants) is mathematically possible, but is unlikely to match the clinical presentation of asymmetric corneal bow tie topography. An alternative approach is to apply vector planning to the second-order Zernike components while also treating other higher order aberrations [19].

One major feature of the method described in this paper is that the laser treatment profile is similar enough to a standard profile that the existing laser nomograms should still apply. This contrasts with generic topography-guided ablations for highly aberrated corneas, which appear to have less predictable refractive astigmatism outcomes [17, 26]. A surgeon using the method in this paper should still be able to accurately target a specific spherocylindrical refractive outcome with a single procedure. The smoothing component of the treatment may cause some slight variation in refractive outcome, depending on the particular smoothing function that is being used, especially if the smoothing extends across an angular domain that is wider than that described in this paper. It is important to note that the overall tissue ablation depth is not affected by smoothing. The smoothing process makes the treatment across the transition zone more even.

The example used in the results section is of an eye with inferior steepening, which is a common type of corneal asymmetry, most extremely found in several earlier stages of keratoconus. In such an eye, the superior part of the cornea may appear to be normal, while the inferior part of the cornea may be misshapen and steep. A uniform treatment across the whole cornea that reduces refractive cylinder alone would effectively apply treatments to the cornea that are not consistent with both the flatter normal-looking part of the cornea and the steeper misshapen portion of the other hemidivision. This unfavorable scenario motivates the use of a customized treatment that is designed to have more effect on areas that are more abnormal and less effect in areas that appear normal. Our use of two different refractive emphases (when applying the vector planning technique to the corneal hemidivisions) allows such specific customization. The steeper inferior corneal hemidivision lends itself to an emphasis towards corneal sphericity, while the more normal superior corneal hemidivision can be treated with a more standard treatment that is closer to refractive cylinder. The emphases for the two hemidivisions need to be chosen by the surgeon by considering postoperative refractive and corneal priorities, including the key parameters of the orientation of the target corneal astigmatism and its magnitude [14].

In the example in this paper with 80% superior refractive emphasis and 50% inferior refractive emphasis, the final target corneal astigmatism after treatment of both hemidivisions is 0.48 D with a steep meridian at 46 degrees. The orientation here appears to be unsatisfactorily oblique. Further analysis shows that the orientation of the target corneal astigmatism must be oblique in this case as it is predetermined by the corneal hemidivisional astigmatisms and the manifest refractive cylinder. The superior hemidivisional corneal target is oriented at 178 degrees and the inferior hemidivisional corneal target is oriented at 62 degrees, so the final overall corneal target steep meridian must lie between these, namely in an arc between 178 and 62 degrees (with the arc extending from 178 to 180 degrees, and then from 0 to 62 degrees). Thus, the example described in this paper does not allow a with-the-rule astigmatism target even though it might be a preferred orientation. Also, if an against-the-rule astigmatism target were preferred, then this would require the vector planning emphasis for the inferior corneal hemidivision to be more targeted more towards corneal sphericity than zero manifest refractive cylinder. These are the planning options available to the surgeon to provide the most favorable corneal and refractive outcome after reducing and regularizing the astigmatism by the maximal amount. Any treatment that does not coincide with the ORA line does not achieve a maximal reduction of astigmatism when considering both corneal and refractive components. The authors are currently refining custom software known as Designer Cornea^®^ that allows surgeons to experiment with different hemidivisional emphases and show the resulting postoperative corneal and refractive targets.

At the moment, it is not possible to perform a treatment using the method proposed in this paper because no excimer laser manufacturer has software that supports such treatment profiles. When such treatments become possible in the future, a clinical evaluation would show how much visual benefit can be gained through such a treatment: a hemidivisional customized laser treatment that both maximally reduces and regularizes the corneal astigmatism to the most favorable orientation, while also reducing the refractive cylinder to the minimum achievable and correcting the spherical ametropia.

## Abbreviations

CorT: Corneal topographic astigmatism
ORA: Ocular residual astigmatism
TIA: Target induced astigmatism vector
